# The DUF221 domain-containing (DDP) genes identification and expression analysis in tomato under abiotic and phytohormone stress

**DOI:** 10.1080/21645698.2021.1962207

**Published:** 2021-08-11

**Authors:** Muhammad Waseem, Mehtab Muhammad Aslam, Iffat Shaheen

**Affiliations:** a College of Horticulture, South China Agricultural University, Guangzhou, China; b College of Life Sciences, Fujian Agriculture and Forestry University, Fuzhou, China; c Faculty of Agriculture Science and Technology, Bahauddin Zakariya University, Multan, Pakistan

**Keywords:** Gene family, miRNA target, expression profile, Duplications, Phylogeny

## Abstract

The domain of unknown function (DUF221 domain-containing) proteins regulates various aspects of plant growth, development, responses to abiotic stresses, and hormone transduction pathways. To understand the role of DDP proteins in tomato, a comprehensive genome-wide analysis was performed in the tomato genome. A total of 12 DDP genes were identified and distributed in 8 chromosomes in the tomato genome. Phylogenetically all SlDDPs were clustered into four clades, subsequently supported by their gene structure and conserved motifs distribution. The SlDDPs contained various cis-acting elements involved in plant responses to abiotic and various phytohormones stresses. The tissue-specific expression profile analysis revealed the constitutive expression of *SlDDPs* in roots, leaves, and developmental phases of fruit. It was found that *SlDDP1, SlDDP3, SlDDP4, SlDDP9, SlDDP10*, and *SlDDP12* exhibited high expression levels in fruits at different development stages. Of these genes, *SlDDP12* contained ethylene (ERE) responsive elements in their promoter regions, suggesting its role in ethylene-dependent fruit ripening. It was found that a single SlDDP induced by two or more abiotic and phytohormone stresses. These include, *SlDDP1, SlDDP2, SlDDP3, SlDDP4, SlDDP7, SlDDP8*, and *SlDDP10* was induced under salt, drought, ABA, and IAA stresses. Moreover, tomato SlDDPs were targeted by multiple miRNA gene families as well. In conclusion, this study predicted that the putative DDP genes might help improve abiotic and phytohormone tolerance in plants, particularly tomato, rice, and other economically important crop plant species.

## Introduction

Plants cope with abiotic and phytohormone stresses in several ways, including physiochemical, morphological, and ultrastructural changes in a cell at various molecular events. Therefore, uncovering the roles of distinct gene families against various stresses helps to identify their particular role.^1^ The availability of the plant genome enables us to identify and characterize various gene families under abiotic and biotic stresses. For instance, several gene families have been identified and characterized, such as NAC,^2,3^ bHLH,^4^ MAPK,^5^ bZIP,^6–8^ GRAS,^9^ Aux/IAA,^10^ TIFY,^11^ FKBP,^12^ PEPC,^13^ and HSF transcription factors.^14^ In addition, plants’ genomes contained plenty of stress-responsive proteins with highly conserved domain^15,16^ and play critical roles in various plant biological processes during stress conditions. Genes with such potential hypothetical domains are classified as a domain of unknown functions (DUF). In recent years, the rapid development of proteomics and genomics identified and sequenced plenty of species’ genomes having enormous DUF superfamilies.^17^ However, there have been some reports of many DUF gene families in plants including DUF221, DUF581, DUF668, DUF724, DUF810, DUF866, DUF936, DUF966, DUF1644, and DUF1618 in rice, *Gossypium hirsutum*, and Arabidopsis.^18–21^

The systematic study of DUF superfamily genes lays the foundation for analyzing these DUF family genes in regulating plant growth and development and tolerance to biotic and abiotic stresses. DUF proteins also function as a membrane protein associated with other related proteins, implying their roles as membrane integral proteins.^22^ The DUF-mediated stress resistance has been reported in only model plants, while comprehensive DUF gene family analysis in other plant species remains determined. For example, *Brassica juncea* dehydration-responsive gene (*ERD4*),^22^ rice drought-responsive gene *AtCSC1* in Arabidopsis,^23^ and its homolog in rice (*OsCA1*) associated with osmotic regulation.^23,24^ DUF538 and DUF27 have the chlorophyll-binding ability^25,26^ and bind to ADP-ribose precisely,^27^ respectively. Arabidopsis DUF283 superfamily is essential for siRNA processing in gene silencing.^28^

DUF genes have also been related to phytohormone and abiotic stress responses, particularly drought and salinity. The *OsSIDP366* and *SIDP361* (DUF1644 superfamily) positively regulate the response to salinity and drought stress in rice.^21,29^ The OsSIDP366 overexpression exhibits more substantial salt tolerance and drought resistance.^29^ Similarly, *SIDP361, OsDSR2*, and *OsDUF810* of DUF1644, DUF966, and OsDUF810 superfamily play a role in dehydration-mediated nutritional status regulation.^20,21,29^ Arabidopsis overexpressing the salt-inducing gene *TaSRHP* of DUF581 superfamily can enhance salinity tolerance and drought resistance.^30^ DUF221 domain-containing proteins (DDP) belong to the anoctamin/calcium-activated chloride channels/ *TMEM16* family.^31^ The DDP proteins play an essential role in plant growth, development, phytohormone signaling, and responses to abiotic and biotic stresses.^32^ This suggesting that DUF domain-containing genes may play a direct or indirect role in pant tolerance.

Tomato (*Solanum lycopersicum*) is an important climacteric vegetable fruit crop and highly sensitive to abiotic stresses^33,34^ affecting plant growth, reduced photosynthesis rate, disrupted ions homeostasis, and tomato productivity.^35,36^ However, the status of this signature domain remains to be determined in tomato. Therefore, taking advantage of DDP putative role in various biological processes, we performed a comprehensive study of DDP gene family in the tomato genome. We predicted *in-silico* subcellular localization, generated an unrooted phylogeny, and analyzed all putative gene expression profiles in different organs/tissues. Additionally, we have performed salinity, drought (PEG), and phytohormone stresses and analyzed the temporal expression profile of SlDDPs. Thus, our data can have the potential to provide a foundation for functional validation of the tomato DUF221 genes and their role in tomato plant growth and development under stressful conditions.

## Material and Method

### Discovery of DDP Gene Family in the Tomato Genome

The tomato whole-genome sequence data were downloaded from the Solanaceae Genomics Network (SGN, https://www.solgenomics.net/).^37^ The Arabidopsis DDP protein sequences were retrieved from the TAIR database (https://www.arabidopsis.org/, Table S1).^38^ The DDP proteins of tomato (SlDDPs) were predicted using a hidden Markov model (HMM) profile retrieved from the Pfam database.^39^ The *S. lycopersicum* DDP protein sequences were searched by using the HMMSEARCH program.^40^ All redundant DDP sequences were excluded. The domains of putative sequences were verified with SMART program^41^ and NCBI CDD.^42^ Sequence Manipulation Suite (SMS)^43^ was used to predict the physicochemical properties of DDP peptide sequences, including molecular weight (MW, kDa), the grand average of hydropathy (GRAVY), and theoretical isoelectric point (pI). For DDP genes nomenclature in tomato, members of the gene family were named 1 to 12 in chronological order on the chromosomes. DDPs chromosomal location was obtained from SNG, and MAP2Chromomse program (v2) was used to visualize each gene on corresponding chromosome.

### In-silico Subcellular Location, Conserved Motif, and Gene Structure of Tomato DDP Genes

The peptide sequences of deduced DDP proteins were submitted to WoLF PSORT program (https://wolfpsort.hgc.jp/)^44^ for *in-silico* protein cellular localization prediction. Tomato DDP protein sequences were scanned in the MEME program (https://meme-suite.org/meme/tools/meme)^45^ to identify conserved motifs with parameters used by Mondal et al.^46^ The gene intron/exon number and distribution were determined in the Gene Structure Display Server (GSDS, http://gsds.gao-lab.org/Gsds_about.php)^47^ by submitting corresponding CDS genomic sequences of SlDDPs.

### Phylogeny and Gene Duplication of SlDDP Genes

The multiple sequence alignment of tomato DDP proteins was performed using Clustal Omega.^48^ An unrooted phylogenetic tree was generated using MEGAX software^49^ by neighbor-joining (NJJ) method^50^ with bootstrap set at 1000 replicates. MCScanX program (https://github.com/wyp1125/MCScanX) was used to predict SlDDP gene duplication events in the tomato genome. The non-synonymous (Ka), synonymous (Ks) nucleotide substitution rates, and the Ka/Ks ratios were predicted using k-estimator (http://en.bio-soft.net/format/KEstimator.html).^51^ The divergence time (T, mya; millions year ago) was calculated as follows: T = Ks/2y (y = 6.56 × 10^−9^).^52^

### Tomato DDP Genes Cis-regulatory Elements, and miRNAs Target Prediction

A 2000bp long 5`UTR nucleotide sequence from the start codon was extracted for each DDP gene from SNG and submitted to PlantCARE database (http://bioinformatics.psb.ugent.be/web tools/plantcare/html/)^48^as query sequence for putative *cis*-regulatory motif prediction. In addition, to predict miRNAs target the putative DDPs, the cDNA sequences of each SlDDPs were submitted to psRNATarget (http://plantgrn.noble.org/psRNATarget/)^53^ against all tomato miRNAs reported in miRbase.^54^

### Plant Growth and Material Collection

The plants of tomato cultivar Micro-Tom have grown in a greenhouse under control conditions: 14 h light /12 h dark photoperiod, at 25°C/20°C day/night temperature with relative humidity between 70% and 80% and photon density of about 120 µmol photons m^−2^s^−^.^1,55^ When tomato seedlings were 6-week-old, different plant parts, including root, leaves, flower (in bud/fully opened), and different developmental phases of fruit (1/2/3 cm, MG; mature green, B; breaker, B10; 10 days breaker) were collected for tissue/organ-specific expression analysis.

In the sixth week, tomato seedlings were treated with 200 mM NaCl, 0.01 mM abscisic acid (ABA), gibberellins (GA3), indole-3-acetic acid (IAA, Auxin), and polyethylene glycol (PEG).^12^ Plants were harvested at 0 h, 3 h, 6 h, 12 h, and 24 h intervals after treatments. Three independent biological replicates were collected, and six seedlings were used for each treatment. All the samples were immediately frozen in liquid nitrogen and stored at −80 °C until further analysis.

### RNA Extraction, cDNA Preparation, and RT-qPCR Analysis

Total RNA extracted from selected samples (tissue-specific/hormone treated) using TRIZOL reagent according to manufacturer’s protocol. The RNA was qualified using nanodrop (Thermo USA), and the quality was assessed through 2% (w/v) gel electrophoresis. The first complementary DNA (cDNA) strand was prepared using Prime Script ™ RT reagent Kit with gDNA Eraser (Takara, JAPAN). Next, SYBR-Premix Ex Taq-II (TliRNaseH Plus) was used to conduct qRT-PCR on CFX96 Touch ™ Real-Time PCR Detection System (BIO-RAD, USA). The housekeeping gene *SlUBQ* (*Solyc01g056940*) was used as an internal control. The relative expression was calculated following 2^−ΔΔCt^ method.^56^ Finally, the heat map was generated using MeV 4.9 software package. All the primers used in this study are listed in Table S2.

## Results

### Identification of DDP Genes in the Tomato Genome

**Figure 1.**
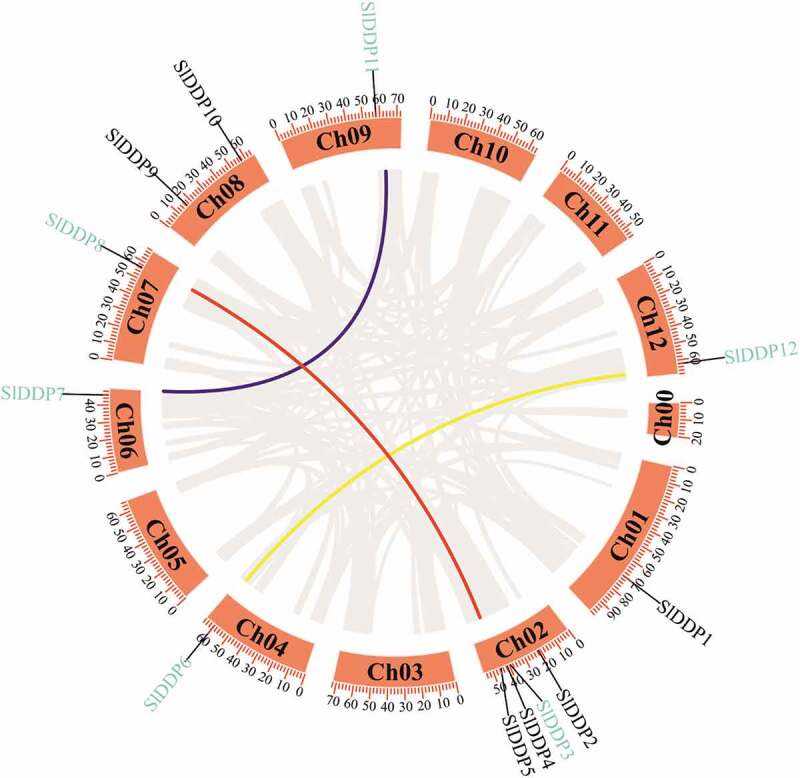
Circos plot showing the physical location of 12 DDP genes distribution in 12 tomato chromosomes, including chromosome 0 (for unallocated genes). The segmental duplication pairs are marked with blue color. The color lines in the circus plot indicate SlDDP segmental duplication between different chromosomes, including *SlDDP3-SlDDP8, SlDDP7-SlDDP11*, and *SlDDP6-SlDDP12*. The scale at the top of each chromosome indicates the size of the chromosome in MBs.

Total 18 DDP genes were identified in the tomato genome using Arabidopsis DDP as a query in the tomato SNG genome. To determine the reliability and validity of putative DDP genes, the protein sequences of identified tomato DDPs were submitted to NCBI and SMART. Finally, 12 unique SlDDP genes (protein sequences are provided in Table S1) were identified and designated as *SlDDP1* to *SlDDP12*. Twelve SlDDP genes were unevenly distributed across the 12 tomato chromosomes. Chromosome 2 contained a comparatively high number of tomato DDPs (four genes). While chromosome 8 contained two genes (*SlDDP9* and *SlDDP10*), chromosomes 1, 4, 6, 7, 9, and 12 had a single gene (Fig. 1). The protein sequences analysis of putative SlDDP genes revealed that the protein length varied from 684 aa (*SlDDP2*) to 977 aa (*SlDDP12*) with MW ranged from 77.07 kDa (*SlDDP11*) to 110.05 kDa (*SlDDP12*). The GRAVY ranged from 0.027 (*SlDDP12)* to 0.383 (*SlDDP5*), and pI varied from 6.7 (*SlDDP4)* to 9.6 (*SlDDP2, SlDDP6*, and *SlDDP7*) suggested that tomato SlDDPs working in a wide range of microenvironment. *In-silico* subcellular location prediction revealed that all the SlDDP associated with the plasma membrane (Table 1).

**Table 1.** Characterization of protein sequences of 12 SlDDP gene family members in tomato genome

Gene ID	Name	aa	MW	pI	GRAVY	Chromosome			Subcellular location prediction
						Number	Start	End	
Solyc01g068500	SlDDP1	705	80.8	8.91	0.264	1	70,131,982	70,142,492	plas:9, E.R.:3, golg:2
Solyc02g036260	SlDDP2	684	78.31	9.6	0.082	2	21,172,510	21,179,750	plas:13, vacu:1
Solyc02g081030	SlDDP3	686	78.35	8.3	0.093	2	39,662,050	39,674,092	plas:8, vacu:2, E.R.:2, cyto:1, mito:1
Solyc02g083430	SlDDP4	831	93.93	6.7	0.127	2	41,421,699	41,424,194	plas:10, E.R.:2, nucl:1, vacu:1
Solyc02g088300	SlDDP5	716	81.1	8.93	0.383	2	45,016,695	45,024,991	plas:13, golg:1
Solyc04g077400	SlDDP6	719	81.45	9.64	0.309	4	59,898,021	59,905,315	plas:10, vacu:2, E.R.:2
Solyc06g084330	SlDDP7	766	88.07	9.64	0.142	6	45,777,999	45,781,628	plas:12, vacu:1, E.R.:1
Solyc07g048110	SlDDP8	796	91.05	9.14	0.181	7	56,620,600	56,630,095	plas:11, vacu:2, E.R.:1
Solyc08g023440	SlDDP9	723	81.95	9.63	0.29	8	16,571,646	16,577,518	plas:12, vacu:1, E.R.:1
Solyc08g076310	SlDDP10	815	94.03	9.46	0.057	8	57,437,506	57,445,845	plas:12, chlo:1, E.R.:1
Solyc09g064810	SlDDP11	673	77.07	9.45	0.129	9	57,811,490	57,819,337	plas:12, vacu:1, E.R.:1
Solyc12g088230	SlDDP12	977	110.05	9.57	0.027	12	62,114,857	62,122,490	plas:8, vacu:3, E.R.:2, golg:1

aa; amino acid, MW; molecular weight, pI; isoelectric point, GRAVY; the grand average of hydropathy, nucl; nucleus, cyto; cytoplasm, chlo; chloroplast, plas; plasma membrane, vacu; vacuole, E.R; endoplasmic reticulum, golg; golgi apparatus

### Phylogenetic Analysis, Gene Structure, and Conserved Motifs Analysis in SlDDPs

**Figure 2.**
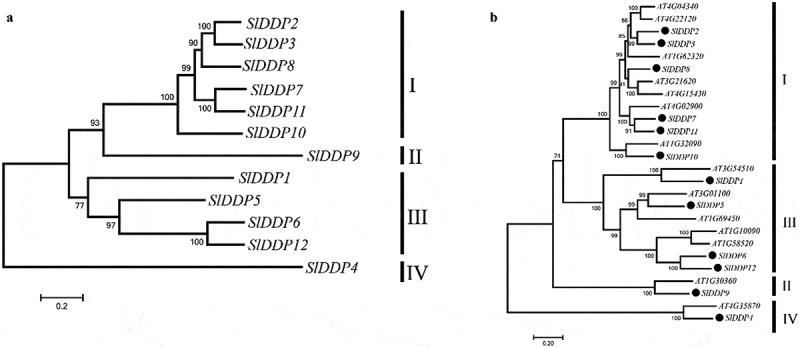
Phylogeny of DDPs gene family. An unrooted neighbor-joining (NJJ) phylogenetic tree of (a) tomato SlDDPs and (b) with *Arabidopsis* DDPs were generated using MEGA 7 program. The bootstrap was set at 1000 replicates. All the DDPs were clustered into four clades named I, II, III, and IV.

To find out peptide sequence conservation in DDPs, tomato DDP protein sequences were aligned. It was observed that DUF221 domain region was highly conserved across all SlDDPs (Fig. S1). To ascertain the phylogenetic relationship among tomato SlDDPs, an unrooted NJJ phylogenetic tree with 12 tomato SlDDPs (Fig. 2a) along with 14 AtDDPs from the Arabidopsis genome (Fig. 2b) was generated. The SlDDPs clustered into four groups (I, II, III, and IV). The SlDDPs pairwise similarity ranges from 26.13 (*SlDDP10/ SlDDP4*) to 84.94% (*SlDDP3/ SlDDP2*). It was found that *SlDDP3* and *SlDDP2* proteins have a similarity index of 89%, clustered together in group I. Similarly, *SlDDP12* and *SlDDP6* were grouped in group III with sequence similarity of 79.13% (Table S3, Fig. 2).

**Figure 3.**
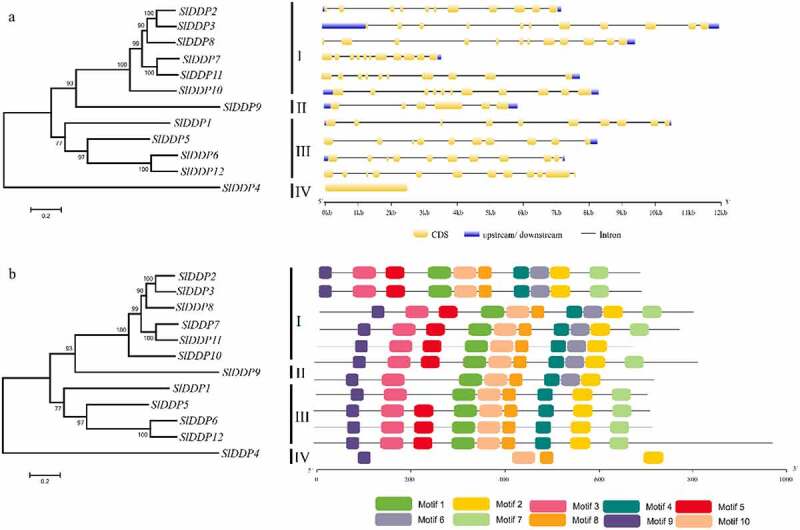
Gene structure analysis of tomato DDPs. (a) The number and distribution of exons and introns and (b) conserved motifs in SlDDPs identified using GSDS and MEME tools, respectively. The scale at the bottom is represented in Kb.

To further gain insight into the structural diversity of SlDDPs genes, intron-exon organization, and conserved motifs numbers and their distribution were analyzed. It was found that the majority of SlDDPs exhibited similar gene structures sharing the same clusters. For instance, SlDDPs contained at least a single exon (*SlDDP4*) and a maximum of eleven exons (genes in phylogenetic cluster I) (Fig. 3a). A total of 10 conserved motifs (Table S4) were identified in SlDDPs consistent with their phylogenetic clustering. All the members of group-I contained 10 motifs; eight motifs are shared by group-II. Similarly, nine and four motifs are present in SlDDPs of group-III and group IV, respectively (Fig. 3b). Taken together, the members of tomato SlDDPs sharing similar gene structure and conserved motifs, implying functional similarly of the SlDDPs within the same group.

### Gene Duplication of SlDDP Genes

Moreover, to elucidate the evolutionary relationship of SlDDP within the tomato genome, synteny analysis was performed. These results revealed that tomato SlDDP sharing three paralogous gene pairs displayed segmental duplication pairs in the whole genome while no tandem duplications were found (Fig. 1). These findings are consistent with the phylogenetic clustering of SlDDP gene. To assess the selection mode of the duplicated SlDDP genes, we estimated the average rate Ka *vs*. Ks by calculating the Ka/Ks ratio for each pair of duplicated SlDDP genes. In general, the Ka/Ks ratio < 1 suggests purifying selection; a ratio = 1 indicates neutral selection, while a ratio >1 indicates that these proteins may have been subject to positive selection. All the three segmental duplicated pairs in the tomato DDP family showed that the Ka/Ks ratios for these duplicated pairs were < 1. Based on the Ka/Ks analyses, we concluded that purifying selection may be primarily responsible for the function maintenance of SlDDP proteins. Based on a substitution rate of 6.5 × 10 − 9 substitutions per site per year, the duplication events for the three segmental duplications were estimated to have occurred approximately between 14.29 and 92.23 mya (Table S5).

### Cis-regulatory Elements in Promoter Sequences of SlDDPs

**Figure 4.**
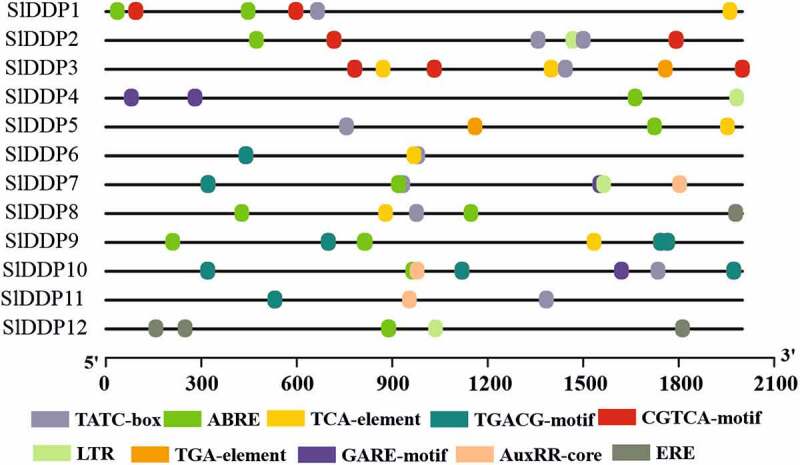
Predicted putative *cis*-regulatory elements in promoter regions of tomato DDP gene family members. TGA-element; Auxin-responsive element, ABRE; *cis*-acting element involved in the abscisic acid responsiveness, CGTCA/TGACG-motif; *cis*-acting regulatory element involved in the MeJA-responsiveness, ERE; Ethylene-responsive element, ABRE; involved in ABA responsiveness, LTR; *cis*-acting element involved in low-temperature responsiveness, AuxRR-core; *cis*-acting regulatory element involved in auxin responsiveness, TATC-box/GARE-motif; gibberellin-responsive element. A promoter sequence up to 2000 bp from 5`UTR was retrieved from SNG and submitted to PlantCARE database for *cis*-regulatory elements prediction.

To investigate the putative role of SlDDPs in plant development under abiotic and biotic stress, we analyzed the promoters of all putative SlDDPs. It was observed that the promoters of SlDDPs contained *cis*-regulatory elements related to plant development, phytohormone, and abiotic stress-responsive elements in their promoter regions. In addition, several phytohormone-related elements including, ABRE, TGA-element (auxin), ERE (ethylene), GARE-motif (gibberellic acid), TGACG-motif (methyl jasmonate), and SARE (salicylic acid), were detected. Furthermore, development-related elements, GCN4-motif, CAAT-box, and several abiotic stress-responsive elements such as MBS, HSE, ARE, TC-rich repeats were also observed (Fig. 4, Table S6).

### miRNAs Targeting the DDP Family Members of the Tomato

To find out miRNAs targeting the SlDDPs, psRNATarget predicted that five SlDDPs gene family members were targeted by conserved miRNA. For instance, *SlDDP3* or *SlDDP5* was targeted by two different miRNAs gene families each. Sly-miRNA395 family (sly-miRNA395a and sly-miRNA395b) and sly-miRNA717 family (sly-miRNA717b), causing cleavage and inhibition of translation of *SlDDP3*. A single member from sly-miR319 family (sly-miR319b) and sly-miR6022 family member target to cleave of *SlDDP5* gene. However, *SlDDP*7, *SlDDP10*, and *SlDDP11* were of sly-miR6026, sly-miR6022, and sly-miR482a family members (Table S7).

### Expression Patterns of SlDDPs in Different Parts of Tomato Plant

**Figure 5.**
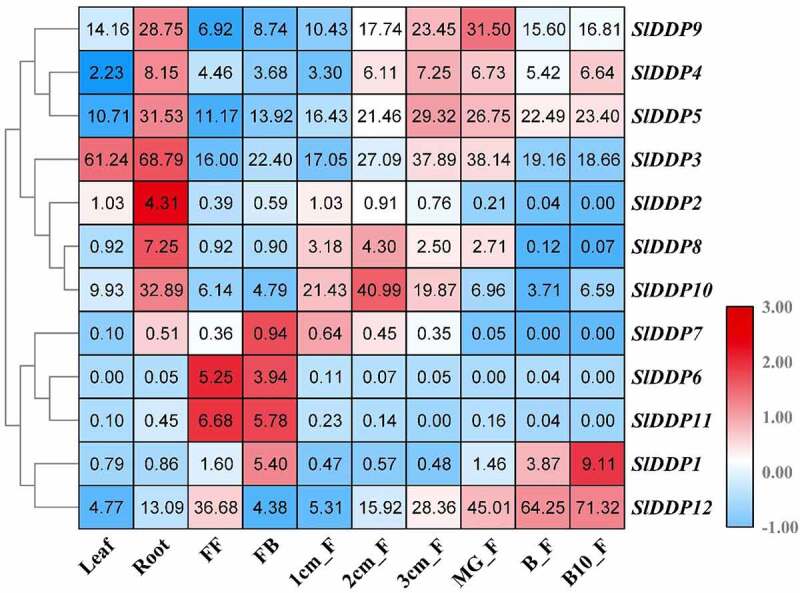
Tissue/organ-specific expression of SlDDPs in tomato. Expression profile of 12 SlDDPs in various plant parts, including root, leaf, flower, and fruits. FF; Fully opened flowers, FB; Flower bud, 1/2/3 cm_F; 1/2/3cm fruit, MG_F; Mature green fruit, B_F; Breaker fruit, and B10_F; ten days Breaker fruit. Heatmap was generated using log^2^ transformed RT-qPCR values.

To predict the biological role of tomato SlDDPs genes, the expression of all the putative SlDDPs were investigated in various plant parts including, roots, leaves, flowers, and fruits at different development stages (Fig. 5). The results revealed that SlDDPs showed significant expression preference with expression higher in specific tissues. These indicated that these SlDDP genes play a role in the development of these tissues. However, few genes were expressed in an only single tissue or plant part. For example, *SlDDP6* and *SlDDP11* expressed with high levels in flower, *SlDDP2* and *SlDDP8* expressed with significant transcript abundance in the root.

Moreover, the genes with more significant transcript abundance include *SlDDP9* in the root, mature green fruit, *SlDDP5* in the root, 3 cm fruit, mature green fruit, *SlDDP3* in leaves and root, *SlDDP10* in root and 2 cm fruit, *SlDDP1* in 10 days breaker fruit and *SlDDP12* I breaker and ten-day breaker fruit. It was observed that some SlDDP genes such as *SlDDP1, SlDDP3, SlDDP4, SlDDP7, SlDDP9*, and *SlDDP10* contained ethylene promoter (ERE, Fig. 4) in their promoter sequences. The expression profile of these genes during various stages of fruit development revealed their elevated transcript abundance except for *SlDDP7*. These suggested that these genes may play an essential role in ethylene-dependent tomato fruit ripening (Fig. 5).

### Phytohormone and Abiotic Stress-inducible Expression Analysis of Putative Tomato DDPs

To further gain insight into the putative role of SlDDPs in tomatoes. We investigated the expression profiles of SlDDPs under two abiotic stresses and three phytohormones, including salt and drought, abscisic acid (ABA), gibberellins (GA3), and auxin (IAA).

**Figure 6.**
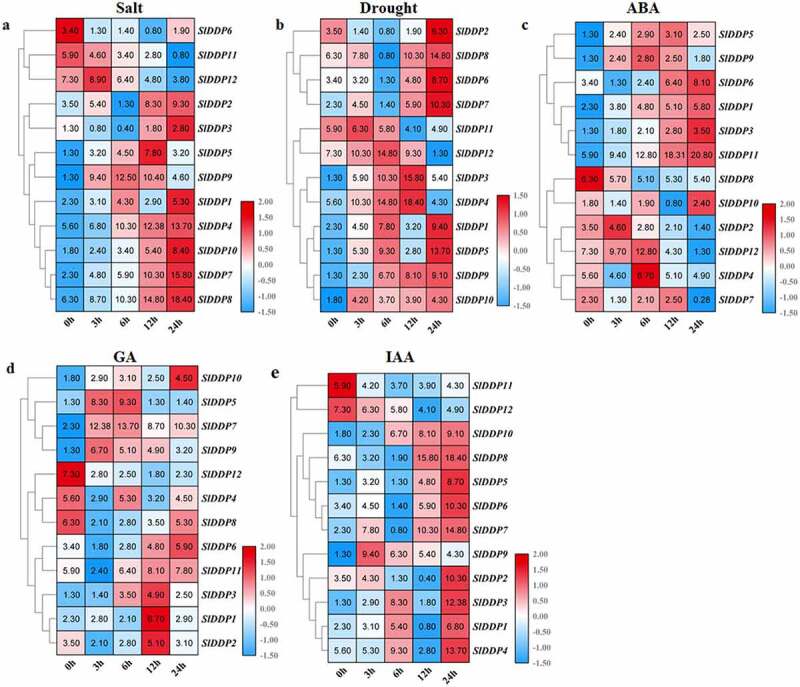
Expression analysis of SLDDPs under salinity, drought, and Phytohormones. Heatmap showing expression profile of 12 SlDDPs under (a) salt, (b) drought (PEG), (c) Abscisic acid (ABA), (d) Gibberellin (GA3), and (e) Auxin (IAA) at 0 h, 3 h, 6 h, 12 h, and 24 h time points. Plants at 0 h time interval were used as control. Heatmap was generated using log^2^ transformed RT-qPCR values.

For salt treatment, most SlDDP genes were upregulated over various time points, but few were downregulated upon exposure. *SlDDP6, SlDDP11*, and *SlDDP12* were downregulated along with all-time points but, *SlDDP1, SlDDP2, SlDDP3, SlDDP4, SlDDP7, SlDDP8*, and *SlDDP10* exhibited opposite trends and were peaked at 24 h interval. Similarly, *SlDDP5* was peaked at 12 h, but *SlDDP9* was peaked at 9 h (Fig. 6a). Under PEG stress, *SlDDP2, SlDDP3, SlDDP6, SlDDP7*, and *SlDDP8* were upregulated at 24 h after stress. *SlDDP11* was upregulated at 3 h time point and then downregulated in subsequent time intervals. *SlDDP12* sharply upregulated till 6 h after treatment and then downregulated in later time points. Similarly, *SlDDP3* and *SlDDP4* were peaked at 12 h and then downregulated at 24 h. the elevated levels of *SlDDP1* and *SlDDP5* were found at 6 h and 24 h time points, but *SlDDP9* was upregulated along with all-time intervals with maximum expressions at 24 h (Fig. 6b).

Under ABA treatment, the transcript levels of *SlDDP1. SlDDP3, SlDDP6*, and *SlDDP11* were increased sharply along with all-time intervals and detected maximum at 24 h. Similarly, the expression levels of *SlDDP4, SlDDP9*, and *SlDDP12* were sharply increased till 6 h and then downregulated in later intervals. *SlDDP10* was peaked at 24 h, but *SlDDP2* showed opposite trends. Moreover, the expression of *SlDDP5* and *SlDDP7* was upregulated till 12 h and then suppressed (Fig. 6c). Under GA treatment, the transcript abundance of *SlDDP6, SlDDP8*, and *SlDDP10* sharply upregulated and peaked at 24 h but, *SlDDP4, SlDDP8, SlDDP9*, and *SlDDP12* was downregulated upon exposure to GA. The expression of *SlDDP6* and *SlDDP11* was significantly upregulated at 12 h and 24 h while *SlDDP1, SlDDP2*, and *SlDDP3* were upregulated at 12 h interval only (Fig. 6d).

For auxin treatment, *SlDDP11* and *SlDDP12* were suppressed but, *SlDDP2, SlDDP5, SlDDP6, SlDDP8*, and *SlDDP10* was peaked at 24 h after treatment. *SlDDP9* was upregulated at 3 h, *SlDDP1, SlDDP3*, and *SlDDP4* were peaked at 6 h and 24 h (Fig. 6e). In comparison, *SlDDP1, SlDDP2, SlDDP3, SlDDP4, SlDDP7, SlDDP8*, and *SlDDP10* was peaked at 24 h under salt, drought, ABA, and IAA stresses. *SlDDP3* was upregulated under drought and GA, *SlDDP9* induced under salt and drought at 6 h and under GA and auxin at 3 h. *SlDDP12* was induced under drought and ABA at 6 h but suppressed in GA and auxin upon exposure. Similarly, *SlDDP8* was suppressed upon treatment, but *SlDDP5* was induced under salt and drought at 12 h, and under drought and auxin at 24 h (Fig. 6a-e).

## Discussion

The plant faces severe destruction from abiotic and biotic stresses during its life cycle, impacting its survival and productivity. Plants have developed tolerance mechanisms to alter their physiology and cellular biochemistry during stresses through changes in gene expression.^57^ Some of these expression products came from genes containing the hypothetical domain of unknown functions (DUF). One such domain is DUF221 and is a highly conserved membrane-associated protein and reported to regulate osmoregulation of calcium through plasma membrane.^23^ To date, the status of DUF221 genes family is mainly unexplored. In this study, DDP gene family was identified under two abiotic (salinity and drought) and three phytohormones (ABA, IAA, GA3) stress, according to previous studies in Arabidopsis,^24^ maize,^58^ and rice.^8,59^

The DDP proteins have been reported to be highly conserved across the plant lineage,^57,60^ which is also confirmed in our study (Fig S1). In this study, 12 DDP genes were identified in the tomato genome and were distributed on 8 chromosomes with a maximum number of genes located on chromosome 2 (Fig. 1). We found that tomato possesses a similar number of DDPs (Table 1) like maize.^58^ There is a loss of three DDPs in tomato compared to Arabidopsis, which possesses the highest number of DPPs 24, whilst one gene is compared to the rice genome.^59^ The number of DDPs in tomato, rice, and maize did not very much, indicating Arabidopsis genome undergo relatively conserved evolutionary history after their divergence. Tomato SlDDPs were clustered into four phylogenetic clades (Fig. 2) as reported previously in Arabidopsis, rice, and maize.^24,58,59^ To further gain insights into the structural changes in DDP genes that occur during the evolution, the gene structure and conserved motifs were analyzed. A high degree of variation in size and distribution of intron and exon was found. The SlDDPs with similar exon and intron were clustered together in a phylogenetic tree. Moreover, it was found in the distribution of conserved motifs (Fig. 3). Similar gene structural diversifications were observed in rice OsDDPs.^8^

Genome duplication plays a pivotal role in speciation and adaptation under various environmental conditions.^61^ The previous study has shown that segmental duplication was primarily responsible for the expansion of DDPs. For instance, four segmental duplications were found in Arabidopsis, and single pair was detected in rice.^57^ In our study, three pairs of SlDDP segmental duplication were found (Fig. 1). Like Arabidopsis, the segmental duplicates of SlDDPs were clustered together into a single clade (clade I and II) (Fig. 2). However, the duplicated pair of rice was found scattered in clade-I and clade-II.^57^ This outcome further substantiates the gene duplication in tomato and Arabidopsis during evolution, which might eventually allow the protein functional diversity by adaptive evolution.^62^ The approximate age of segmentally duplicated DDP (OsDDP3-OsDDP10) paralogues of rice is 64.2 MYA. In contrast, 29, 65, and 71 MYA of gene pairs *AtDDP9-AtDDP13, AtDDP2-AtDDP14*, and *AtDDP12-AtDDP6* indicated that duplication of these pairs could have occurred before the appearance of Poaceae from the common ancestor ~55–70 mya^63^ or crucifers~24–40 mya,^64^ respectively. We found that tomato segmentally duplication DDPs (*SlDDP3-SlDDP8, SlDDP6-SlDDP12*, and *SlDDP7-SlDDP11*) indicated duplications of SlDDPs occurred before divergence Solanaceae from common ancestors about 50–52 mya.^65^

It was reported that rice OsDDPs were regulated by multiple miRNAs. For instance, OsDDP6 was targeted by multiple miRNA families belonging to the osa-miR818 family and osa-miR1436. Similarly, *OsDDP10* is targeted by miRNA osa-miR6248.^8^ Barley miR818^66^ and rice osa-miR1436^66^ and osa-miR6248^67^ have been differentially regulated under drought, salinity, and arsenate stresses. It was found that tomato SlDDPs were also targeted by multi miRNA gene families (Table 73). For example, tomato *SlDDP3* was targeted by sly-miRNA395a, sly-miRNA395, and sly-miRNA717b while sly-miR319b and sly-miR6022 target to cleavage tomato *SlDDP5*. These findings are suggesting that multiple miRNAs may regulate a single gene. *Cis*-regulatory elements play a pivotal role in controlling various aspects of plant growth and development under normal, abiotic, biotic, and phytochrome responses by regulating gene expression. Several *cis*-acting sequences related to phytohormone responses such as ERE, ABRE, GARE, AuxRR-core, TGA elements, abiotic stress-responsive such as HSE, MBS, and MYB were identified in promoter region of SlDDPs (Fig. 4, Table S6).

To our knowledge, although the relationship between AtDDP and ZmDDP proteins and stresses has been reported.^58,68^ The dynamic abiotic and phytohormone-responsive expression patterns of SlDDPs were still obscure. Expression pattern analysis of SlDDPs helped us to understand their possible functions and offer a thorough foundation for future functional studies. To provide the further foundation for functional characterization of tomato SlDDPs, expression profile analysis under drought, salinity, and phytohormone were evaluated at various time points. It was found that the majority of genes were induced under these stresses while few were downregulated. Ding et al.,^58^ showed that 12 of six ZmOSCAs were significantly upregulated, and expression of single ZmOSCA was down-regulated. The relative expression levels of *OsOSCA1.1, −1.2, −2.1, −2.4, −2.5*, and *−4.1* were upregulated by PEG treatment.^59^ Interestingly, in this study, we found that *SlDDP6, SlDDP11*, and *SlDDP12* were suppressed under salinity, but *SlDDP6* was upregulated under drought stress (Fig. 6a-b), indicating that these genes might serve as key mediators of drought stress responses. *SlDDP4, SlDDP8*, and *SlDDP12* were suppressed under GA and IAA stresses, but *SlDDP4* was upregulated under IAA along with *SlDDP2, SlDDP3, SlDDP4, SlDDP5, SlDDP6, SlDDP7, SlDDP8*, and *SlDDP10* (Fig. 6d-e). *SlDDP1, SlDDP3, SlDDP6*, and *SlDDP11* were upregulated under ABA and IAA (Fig. 6c and Fig.6e). Moreover, *SlDDP2* was upregulated under salinity, drought, GA, and IAA but suppressed under ABA, while *SlDDP8* was suppressed under GA. This induced expression of SlDDPs under various stresses suggested that these genes may involve in multiple stress responses in the tomato plant.

## Conclusion

In summary, a total of 12 tomato SlDDPs genes were identified in the whole genome. Gene structure, conserved motifs, and *cis*-regulatory elements prediction, and phylogeny were analyzed. The expression profile in various parts of the tomato plant was investigated to clue the possible biological and development role of these genes. However, differential abiotic (salinity and drought) and phytohormone inducible expression profile revealed their putative roles in abiotic and hormone transduction pathways. Furthermore, the prediction of miRNAs targets revealed that multiple-miRNAs regulate the expression of tomato SlDDPs. Together, our study will provide helpful information for further functional analysis of DDP genes in tomato and other related plant species.

## Supplementary Material

Supplemental Material

## Abbreviations

DDP, DUF221 domain-containing; MW, molecular weight; kDa, kilo Dalton; GRAVY, the grand average of hydropathy; pI, theoretical isoelectric point; ABA, abscisic acid; GA3, gibberellins; IAA, indole-3-acetic acid; PEG, polyethylene glycol; mM, Millimolar
