# Supplementary material for: The DUF221 domain-containing (DDP) genes identification and expression analysis in tomato under abiotic and phytohormone stress
Source: GM Crops Food. 2021 Aug 11;12(1):586–99. doi: 10.1080/21645698.2021.1962207 (PMC8820248; doi:10.1080/21645698.2021.1962207)
Supplement: Supplemental Material [file KGMC_A_1962207_SM7381.zip › supplementary/Table S2.docx]

**Table S2. List of primers used in this study**

| **Gene** | **Forward** | **Reverse** | **Product size** |
| --- | --- | --- | --- |
| *SlDDP1* | AGCATCTGGATAAGCTTCTAGC | GTCACAAAAGCCACAGGCAA | 202 |
| *SlDDP2* | TGGTTGACTGATTGGGCTCC | AACTTTCAGGAACGGCGCTA | 201 |
| *SlDDP3* | CGTTGCTTGCATGGGCTATC | CAGAGGCGACAAACTGCAAC | 234 |
| *SlDDP4* | AGATGTGGCTGAACTGGGTG | TAGCTCAGCCCTTTGCTCAC | 173 |
| *SlDDP5* | ATCGAGAAAGGTTTGGGCGT | TGCTGCATGTGTGTAGCTGA | 202 |
| *SlDDP6* | GGATGATTGTTTTCAGTTTCCGA | CCCAAAGCCATCTTGACCCT | 171 |
| *SlDDP7* | TTATGGGCACACGTGGTGAT | CATCAGGATCTGGTGGGACG | 169 |
| *SlDDP8* | TGTCATGCGTTCCTTGGTGT | TGTACCCGAGTGCAAAGGAC | 232 |
| *SlDDP9* | TGAATCCGGTTGAAGGTGGG | TTGCTGGTGGTGTTGACTGT | 235 |
| *SlDDP10* | GCTCTGCTTAGACTTCAGCCA | AGCTTGAGGCATCCAGTTGA | 162 |
| *SlDDP11* | GACAGCAGGCTACAGATCCC | ACCATTGCATCCTGATGGCG | 172 |
| *SlDDP12* | TCGGGTACGAACAAGACAGC | AGAGGCACCACGAGGAAAAG | 207 |
| *SlUBQ* | CACCAAGCCAAAGAAGATCAAGC | TCAGCATTAGGGCACTCCTTACG | 200 |
