# Supplementary material for: The DUF221 domain-containing (DDP) genes identification and expression analysis in tomato under abiotic and phytohormone stress
Source: GM Crops Food. 2021 Aug 11;12(1):586–99. doi: 10.1080/21645698.2021.1962207 (PMC8820248; doi:10.1080/21645698.2021.1962207)
Supplement: Supplemental Material [file KGMC_A_1962207_SM7381.zip › supplementary/Table S3.docx]

| **Table S3. Percent similarity matrix showing the percent identity for tomato DDP proteins.** | | | | | | | | |  |  |  |  |
| --- | --- | --- | --- | --- | --- | --- | --- | --- | --- | --- | --- | --- |
|  |  |  |  |  |  |  |  |  |  |  |  |  |
| SlDDP4 | 100% |  |  |  |  |  |  |  |  |  |  |  |
| SlDDP9 | 30.98% | 100% |  |  |  |  |  |  |  |  |  |  |
| SlDDP10 | 26.13% | 44.53% | 100% |  |  |  |  |  |  |  |  |  |
| SlDDP7 | 28.72% | 43.15% | 69.97% | 100% |  |  |  |  |  |  |  |  |
| SlDDP11 | 29.71% | 39.82% | 69.68% | 83.95% | 100% |  |  |  |  |  |  |  |
| SlDDP8 | 28.51% | 42.87% | 69.59% | 75.97% | 74.29% | 100% |  |  |  |  |  |  |
| SlDDP2 | 29.09% | 43.56% | 70.61% | 74.41% | 64.93% | 79.67% | 100% |  |  |  |  |  |
| SlDDP3 | 29.15% | 41.83% | 70.84% | 72.74% | 63.89% | 82.21% | 84.94% | 100% |  |  |  |  |
| SlDDP1 | 29.92% | 39.85% | 45.53% | 47.51% | 42.94% | 45.24% | 44.73% | 43.29% | 100% |  |  |  |
| SlDDP5 | 31.98% | 43.15% | 47.62% | 46.64% | 42.49% | 46.78% | 44.73% | 44.46% | 51.48% | 100% |  |  |
| SlDDP6 | 30.18% | 36.16% | 42.14% | 42.42% | 39.52% | 42.55% | 41.37% | 40.08% | 47.37% | 55.16% | 100% |  |
| SlDDP12 | 27.07% | 37.34% | 40.12% | 41.38% | 40.86% | 40.32% | 42.69% | 42.12% | 46.80% | 56.14% | 79.13% | 100% |
| **Genes** | SlDDP4 | SlDDP9 | SlDDP10 | SlDDP7 | SlDDP11 | SlDDP8 | SlDDP2 | SlDDP3 | SlDDP1 | SlDDP5 | SlDDP6 | SlDDP12 |
