# Supplementary material for: The DUF221 domain-containing (DDP) genes identification and expression analysis in tomato under abiotic and phytohormone stress
Source: GM Crops Food. 2021 Aug 11;12(1):586–99. doi: 10.1080/21645698.2021.1962207 (PMC8820248; doi:10.1080/21645698.2021.1962207)
Supplement: Supplemental Material [file KGMC_A_1962207_SM7381.zip › supplementary/Table S4.docx]

**Table S4.** Putative motifs identified from SlDDP proteins using MEME. The sequence logos were generated using WebLogo.

| **Motif name** | **Sequence logo** | **E-value** |
| --- | --- | --- |
| **Motif 1** | 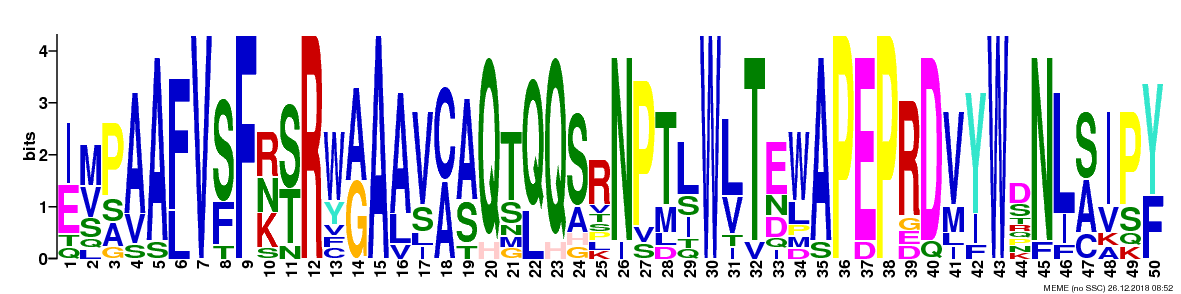  EMPAAFVSFRSRWAAAVCAQTQQSRNPTLWLTEWAPEPRDVYWDNLSIPY | 1.6e-254 |
| **Motif 2** | 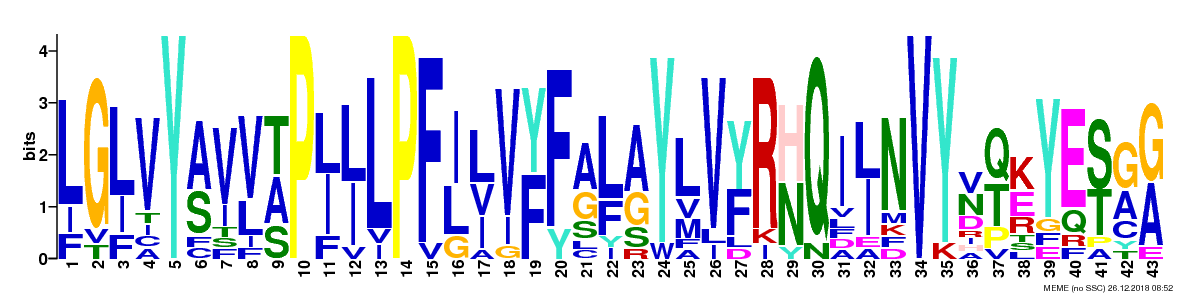  LGLVYAVVTPJJLPFJLVYFALAYLVYRHQIJNVYNQKYESGG | 2.6e-168 |
| **Motif 3** | 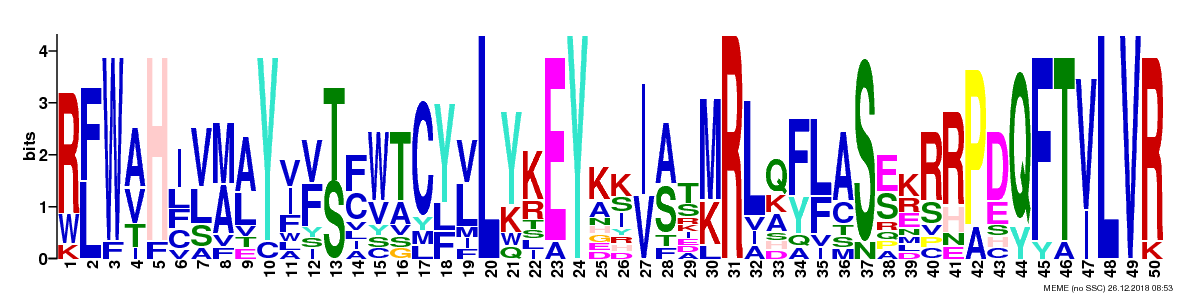  RFWAHJVMAYIVTFWTCYLLYKEYKKIATMRLQFLASEKRRPDQFTVLVR | 4.5e-190 |
| **Motif 4** | 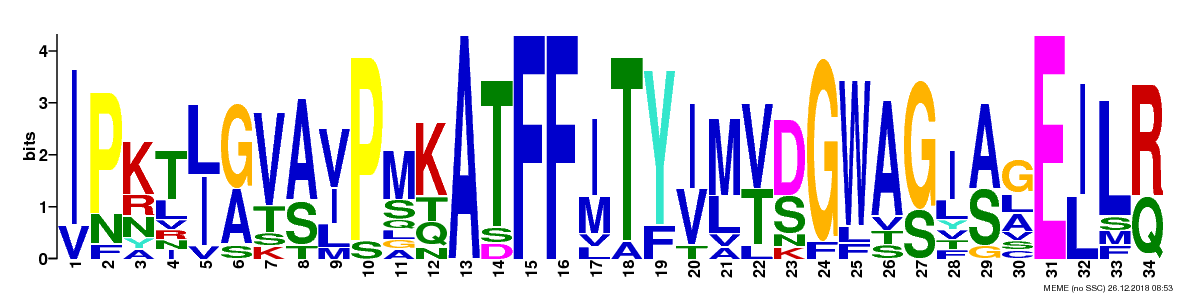  IPKTJGVAVPMKATFFITYIMVDGWAGIAGEJLR | 9.6e-127 |
| **Motif 5** | 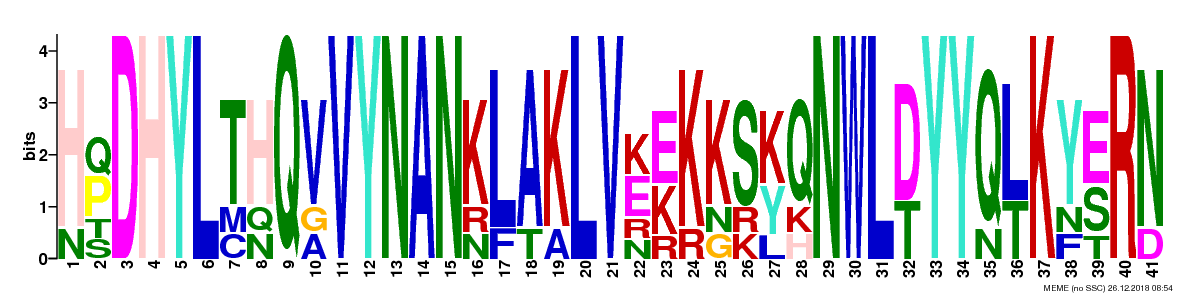  HPDHYLTHQVVYNANKLAKLVKEKKSKQNWLDYYQLKYERN | 4.3e-116 |
| **Motif 6** | 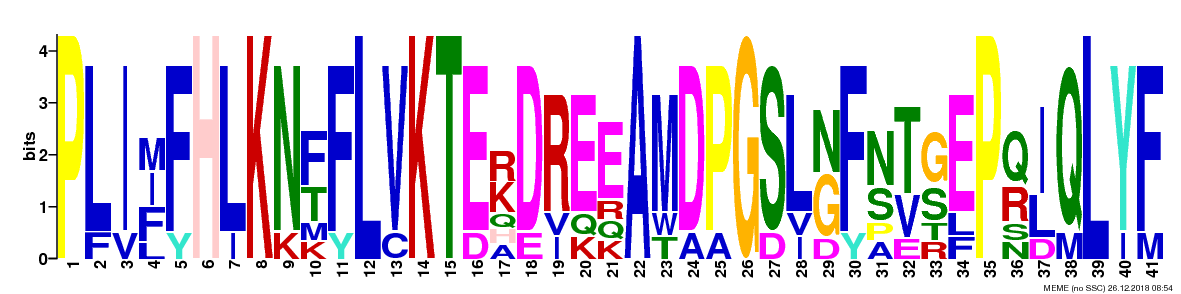  PLIIFHLKNFFLVKTEKDREEAMDPGSLGFNTGEPQIQLYF | 4.2e-106 |
| **Motif 7** | 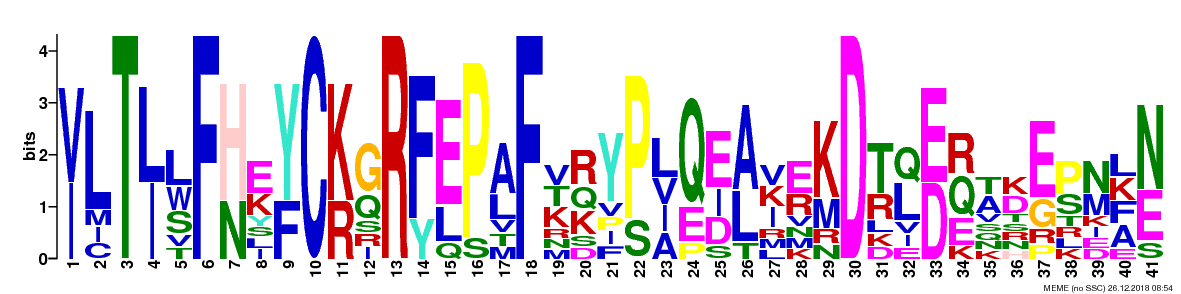  VLTJLFHEYCKGRFEPAFKRYPLQEAIEKDTLERTKEPNKN | 2.8e-092 |
| **Motif 8** | 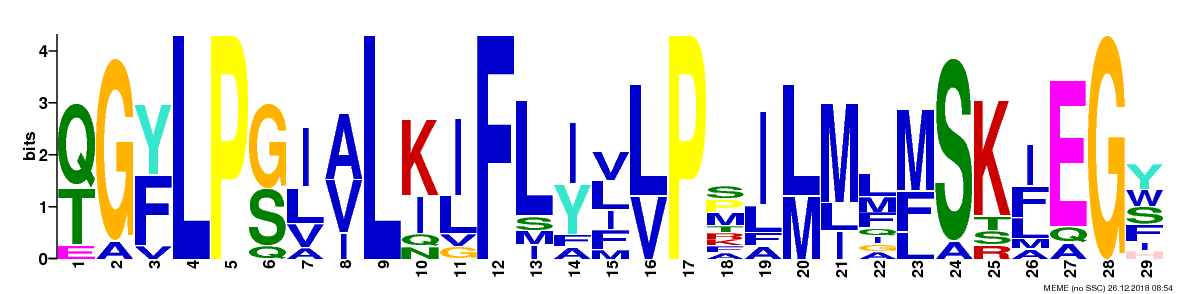  QGYLPGIALKIFLILLPSILMLMSKIEGY | 5.9e-088 |
| **Motif 9** | 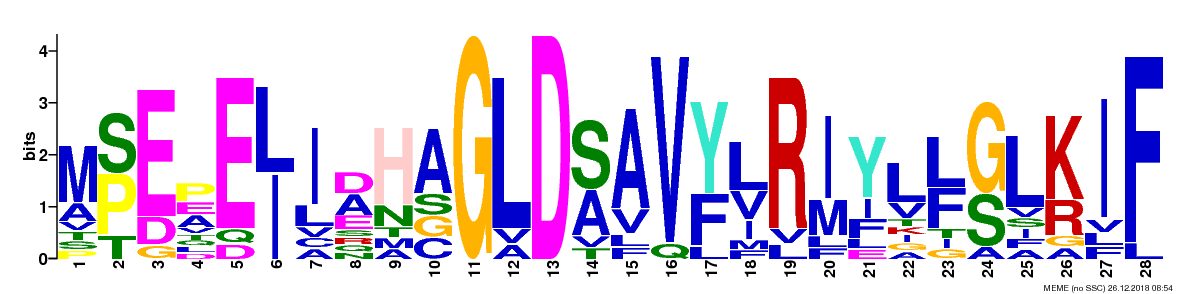  MPEPEJIAHAGLDSAVYLRIYLLGLKIF | 1.4e-082 |
| **Motif 10** | 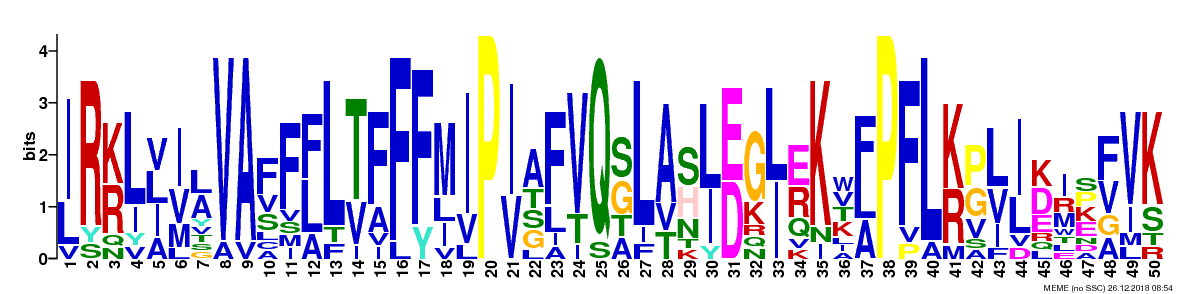  IRKLIIAVAFFFLTFFFMIPIAFVQSLASJEGJEKVFPFLKPLIKIEFVK | 9.1e-121 |
