# Supplementary material for: The DUF221 domain-containing (DDP) genes identification and expression analysis in tomato under abiotic and phytohormone stress
Source: GM Crops Food. 2021 Aug 11;12(1):586–99. doi: 10.1080/21645698.2021.1962207 (PMC8820248; doi:10.1080/21645698.2021.1962207)
Supplement: Supplemental Material [file KGMC_A_1962207_SM7381.zip › supplementary/Table S5.docx]

**Table S5. Ka/Ks analysis and estimated divergence time for segmental duplicated SlDDP genes.**

| **Seq_1** | **Seq_2** | **Ka** | **Ks** | **Ka_Ks** | **Time (Mya*)** | **Purify Selection** |
| --- | --- | --- | --- | --- | --- | --- |
| SlDDP3 | SlDDP8 | 0.182 | 1.901 | 0.096 | 14.29 | Yes |
| SlDDP6 | SlDDP12 | 0.171 | 0.613 | 0.279 | 92.23 | Yes |
| SlDDP7 | SlDDP11 | 0.131 | 0.501 | 0.26 | 75.37 | Yes |

***Mya; millions year ago**
