# Supplementary material for: The DUF221 domain-containing (DDP) genes identification and expression analysis in tomato under abiotic and phytohormone stress
Source: GM Crops Food. 2021 Aug 11;12(1):586–99. doi: 10.1080/21645698.2021.1962207 (PMC8820248; doi:10.1080/21645698.2021.1962207)
Supplement: Supplemental Material [file KGMC_A_1962207_SM7381.zip › supplementary/Table S6.docx]

**Table S6. *Cis-*regulatory elements predicted in SlDDPs**

| **Gene ID** | **Gene name** | **Promoter** | **Sequence** | **Start** | **End** | **Length** | **Strand** | **Plant** | **Description** |
| --- | --- | --- | --- | --- | --- | --- | --- | --- | --- |
| Solyc01g068500 | SlDDP1 | ABRE | ACGTG | 13 | 18 | 5 | - | *Arabidopsis thaliana* | *cis-*acting element involved in the abscisic acid responsiveness |
| Solyc01g068500 | SlDDP1 | ABRE | ACGTG | 424 | 429 | 5 | - | *Arabidopsis thaliana* | *cis-*acting element involved in the abscisic acid responsiveness |
| Solyc01g068500 | SlDDP1 | TCA-element | CCATCTTTTT | 1938 | 1947 | 9 | + | *Nicotiana tabacum* | *cis-*acting element involved in salicylic acid responsiveness |
| Solyc01g068500 | SlDDP1 | P-box | CCTTTTG | 726 | 733 | 7 | + | *Oryza sativa* | gibberellin-responsive element |
| Solyc01g068500 | SlDDP1 | TGACG-motif | TGACG | 71 | 76 | 5 | - | *Hordeum vulgare* | *cis-*acting regulatory element involved in the MeJA-responsiveness |
| Solyc01g068500 | SlDDP1 | TGACG-motif | TGACG | 574 | 579 | 5 | + | *Hordeum vulgare* | *cis-*acting regulatory element involved in the MeJA-responsiveness |
| Solyc01g068500 | SlDDP1 | CGTCA-motif | CGTCA | 71 | 76 | 5 | + | *Hordeum vulgare* | *cis-*acting regulatory element involved in the MeJA-responsiveness |
| Solyc01g068500 | SlDDP1 | CGTCA-motif | CGTCA | 574 | 579 | 5 | - | *Hordeum vulgare* | *cis-*acting regulatory element involved in the MeJA-responsiveness |
| Solyc01g068500 | SlDDP1 | TATC-box | TATCCCA | 642 | 649 | 7 | - | *Oryza sativa* | *cis-*acting element involved in gibberellin-responsiveness |
| Solyc02g036260 | SlDDP2 | TGACG-motif | TGACG | 694 | 699 | 5 | - | *Hordeum vulgare* | *cis-*acting regulatory element involved in the MeJA-responsiveness |
| Solyc02g036260 | SlDDP2 | TGACG-motif | TGACG | 1769 | 1774 | 5 | - | *Hordeum vulgare* | *cis-*acting regulatory element involved in the MeJA-responsiveness |
| Solyc02g036260 | SlDDP2 | LTR | CCGAAA | 1444 | 1450 | 6 | - | *Hordeum vulgare* | *cis-*acting element involved in low-temperature responsiveness |
| Solyc02g036260 | SlDDP2 | ABRE | ACGTG | 450 | 455 | 5 | + | *Arabidopsis thaliana* | *cis-*acting element involved in the abscisic acid responsiveness |
| Solyc02g036260 | SlDDP2 | CGTCA-motif | CGTCA | 694 | 699 | 5 | + | *Hordeum vulgare* | *cis-*acting regulatory element involved in the MeJA-responsiveness |
| Solyc02g036260 | SlDDP2 | CGTCA-motif | CGTCA | 1769 | 1774 | 5 | + | *Hordeum vulgare* | *cis-*acting regulatory element involved in the MeJA-responsiveness |
| Solyc02g036260 | SlDDP2 | TATC-box | TATCCCA | 1335 | 1342 | 7 | + | *Oryza sativa* | *cis-*acting element involved in gibberellin-responsiveness |
| Solyc02g036260 | SlDDP2 | TATC-box | TATCCCA | 1477 | 1484 | 7 | + | *Oryza sativa* | *cis-*acting element involved in gibberellin-responsiveness |
| Solyc02g081030 | SlDDP3 | TGA-element | AACGAC | 1735 | 1741 | 6 | - | *Brassica oleracea* | auxin-responsive element |
| Solyc02g081030 | SlDDP3 | TCA-element | CCATCTTTTT | 848 | 857 | 9 | - | *Nicotiana tabacum* | *cis-*acting element involved in salicylic acid responsiveness |
| Solyc02g081030 | SlDDP3 | TCA-element | CCATCTTTTT | 1376 | 1385 | 9 | - | *Nicotiana tabacum* | *cis-*acting element involved in salicylic acid responsiveness |
| Solyc02g081030 | SlDDP3 | P-box | CCTTTTG | 504 | 511 | 7 | + | *Oryza sativa* | gibberellin-responsive element |
| Solyc02g081030 | SlDDP3 | TGACG-motif | TGACG | 759 | 764 | 5 | + | *Hordeum vulgare* | *cis-*acting regulatory element involved in the MeJA-responsiveness |
| Solyc02g081030 | SlDDP3 | TGACG-motif | TGACG | 1009 | 1014 | 5 | - | *Hordeum vulgare* | *cis-*acting regulatory element involved in the MeJA-responsiveness |
| Solyc02g081030 | SlDDP3 | TGACG-motif | TGACG | 1977 | 1982 | 5 | + | *Hordeum vulgare* | *cis-*acting regulatory element involved in the MeJA-responsiveness |
| Solyc02g081030 | SlDDP3 | TATC-box | TATCCCA | 1421 | 1428 | 7 | + | *Oryza sativa* | *cis-*acting element involved in gibberellin-responsiveness |
| Solyc02g081030 | SlDDP3 | CGTCA-motif | CGTCA | 759 | 764 | 5 | - | *Hordeum vulgare* | *cis-*acting regulatory element involved in the MeJA-responsiveness |
| Solyc02g081030 | SlDDP3 | CGTCA-motif | CGTCA | 1009 | 1014 | 5 | + | *Hordeum vulgare* | *cis-*acting regulatory element involved in the MeJA-responsiveness |
| Solyc02g081030 | SlDDP3 | CGTCA-motif | CGTCA | 1977 | 1982 | 5 | - | *Hordeum vulgare* | *cis-*acting regulatory element involved in the MeJA-responsiveness |
| Solyc02g083430 | SlDDP4 | GARE-motif | TCTGTTG | 57 | 64 | 7 | - | *Brassica oleracea* | gibberellin-responsive element |
| Solyc02g083430 | SlDDP4 | GARE-motif | TCTGTTG | 257 | 264 | 7 | - | *Brassica oleracea* | gibberellin-responsive element |
| Solyc02g083430 | SlDDP4 | ABRE | ACGTG | 1640 | 1645 | 5 | + | *Arabidopsis thaliana* | *cis-*acting element involved in the abscisic acid responsiveness |
| Solyc02g083430 | SlDDP4 | LTR | CCGAAA | 1959 | 1965 | 6 | + | *Hordeum vulgare* | *cis-*acting element involved in low-temperature responsiveness |
| Solyc02g088300 | SlDDP5 | TATC-box | TATCCCA | 733 | 740 | 7 | + | *Oryza sativa* | *cis-*acting element involved in gibberellin-responsiveness |
| Solyc02g088300 | SlDDP5 | TCA-element | CCATCTTTTT | 1930 | 1939 | 9 | + | *Nicotiana tabacum* | *cis-*acting element involved in salicylic acid responsiveness |
| Solyc02g088300 | SlDDP5 | ABRE | ACGTG | 1701 | 1706 | 5 | + | *Arabidopsis thaliana* | *cis-*acting element involved in the abscisic acid responsiveness |
| Solyc02g088300 | SlDDP5 | TGA-element | AACGAC | 1137 | 1143 | 6 | - | *Brassica oleracea* | auxin-responsive element |
| Solyc04g077400 | SlDDP6 | TATC-box | TATCCCA | 957 | 964 | 7 | + | *Oryza sativa* | *cis-*acting element involved in gibberellin-responsiveness |
| Solyc04g077400 | SlDDP6 | CGTCA-motif | CGTCA | 417 | 422 | 5 | - | *Hordeum vulgare* | *cis-*acting regulatory element involved in the MeJA-responsiveness |
| Solyc04g077400 | SlDDP6 | TCA-element | TCAGAAGAGG | 945 | 954 | 9 | - | *Brassica oleracea* | *cis-*acting element involved in salicylic acid responsiveness |
| Solyc04g077400 | SlDDP6 | TGACG-motif | TGACG | 417 | 422 | 5 | + | *Hordeum vulgare* | *cis-*acting regulatory element involved in the MeJA-responsiveness |
| Solyc06g084330 | SlDDP7 | GARE-motif | TCTGTTG | 1529 | 1536 | 7 | - | *Brassica oleracea* | gibberellin-responsive element |
| Solyc06g084330 | SlDDP7 | CGTCA-motif | CGTCA | 298 | 303 | 5 | - | *Hordeum vulgare* | *cis-*acting regulatory element involved in the MeJA-responsiveness |
| Solyc06g084330 | SlDDP7 | TATC-box | TATCCCA | 910 | 917 | 7 | - | *Oryza sativa* | *cis-*acting element involved in gibberellin-responsiveness |
| Solyc06g084330 | SlDDP7 | ABRE | CACGTG | 896 | 902 | 6 | + | *Arabidopsis thaliana* | *cis-*acting element involved in the abscisic acid responsiveness |
| Solyc06g084330 | SlDDP7 | ABRE | ACGTG | 897 | 902 | 5 | + | *Arabidopsis thaliana* | *cis-*acting element involved in the abscisic acid responsiveness |
| Solyc06g084330 | SlDDP7 | LTR | CCGAAA | 1541 | 1547 | 6 | - | *Hordeum vulgare* | *cis-*acting element involved in low-temperature responsiveness |
| Solyc06g084330 | SlDDP7 | TGACG-motif | TGACG | 298 | 303 | 5 | + | *Hordeum vulgare* | *cis-*acting regulatory element involved in the MeJA-responsiveness |
| Solyc06g084330 | SlDDP7 | AuxRR-core | GGTCCAT | 1780 | 1787 | 7 | - | *Nicotiana tabacum* | *cis-*acting regulatory element involved in auxin responsiveness |
| Solyc07g048110 | SlDDP8 | TATC-box | TATCCCA | 953 | 960 | 7 | - | *Oryza sativa* | *cis-*acting element involved in gibberellin-responsiveness |
| Solyc07g048110 | SlDDP8 | ERE | ATTTCATA | 1956 | 1964 | 8 | - | *Nicotiana glutinos* | Ethylene Responsive |
| Solyc07g048110 | SlDDP8 | P-box | CCTTTTG | 160 | 167 | 7 | - | *Oryza sativa* | gibberellin-responsive element |
| Solyc07g048110 | SlDDP8 | TCA-element | CCATCTTTTT | 856 | 865 | 9 | - | *Nicotiana tabacum* | *cis-*acting element involved in salicylic acid responsiveness |
| Solyc07g048110 | SlDDP8 | ABRE | ACGTG | 404 | 409 | 5 | + | *Arabidopsis thaliana* | *cis-*acting element involved in the abscisic acid responsiveness |
| Solyc07g048110 | SlDDP8 | ABRE | ACGTG | 1124 | 1129 | 5 | + | *Arabidopsis thaliana* | *cis-*acting element involved in the abscisic acid responsiveness |
| Solyc08g023440 | SlDDP9 | CGTCA-motif | CGTCA | 676 | 681 | 5 | + | *Hordeum vulgare* | *cis-*acting regulatory element involved in the MeJA-responsiveness |
| Solyc08g023440 | SlDDP9 | CGTCA-motif | CGTCA | 1720 | 1725 | 5 | - | *Hordeum vulgare* | *cis-*acting regulatory element involved in the MeJA-responsiveness |
| Solyc08g023440 | SlDDP9 | CGTCA-motif | CGTCA | 1742 | 1747 | 5 | + | *Hordeum vulgare* | *cis-*acting regulatory element involved in the MeJA-responsiveness |
| Solyc08g023440 | SlDDP9 | TCA-element | CCATCTTTTT | 1511 | 1520 | 9 | + | *Nicotiana tabacum* | *cis-*acting element involved in salicylic acid responsiveness |
| Solyc08g023440 | SlDDP9 | O2-site | GTTGACGTGA | 136 | 145 | 9 | - | *Zea mays* | *cis-*acting regulatory element involved in zein metabolism regulation |
| Solyc08g023440 | SlDDP9 | TGACG-motif | TGACG | 676 | 681 | 5 | - | *Hordeum vulgare* | *cis-*acting regulatory element involved in the MeJA-responsiveness |
| Solyc08g023440 | SlDDP9 | TGACG-motif | TGACG | 1720 | 1725 | 5 | + | *Hordeum vulgare* | *cis-*acting regulatory element involved in the MeJA-responsiveness |
| Solyc08g023440 | SlDDP9 | TGACG-motif | TGACG | 1742 | 1747 | 5 | - | *Hordeum vulgare* | *cis-*acting regulatory element involved in the MeJA-responsiveness |
| Solyc08g023440 | SlDDP9 | P-box | CCTTTTG | 347 | 354 | 7 | - | *Oryza sativa* | gibberellin-responsive element |
| Solyc08g023440 | SlDDP9 | ABRE | ACGTG | 187 | 192 | 5 | + | *Arabidopsis thaliana* | *cis-*acting element involved in the abscisic acid responsiveness |
| Solyc08g023440 | SlDDP9 | ABRE | GACACGTGGC | 789 | 798 | 9 | - | *Triticum aestivum* | *cis-*acting element involved in the abscisic acid responsiveness |
| Solyc08g023440 | SlDDP9 | ABRE | CACGTG | 791 | 797 | 6 | + | *Arabidopsis thaliana* | *cis-*acting element involved in the abscisic acid responsiveness |
| Solyc08g023440 | SlDDP9 | ABRE | ACGTG | 792 | 797 | 5 | + | *Arabidopsis thaliana* | *cis-*acting element involved in the abscisic acid responsiveness |
| Solyc08g076310 | SlDDP9 | GARE-motif | TCTGTTG | 1597 | 1604 | 7 | + | *Brassica oleracea* | gibberellin-responsive element |
| Solyc08g076310 | SlDDP10 | TATC-box | TATCCCA | 1712 | 1719 | 7 | + | *Oryza sativa* | *cis-*acting element involved in gibberellin-responsiveness |
| Solyc08g076310 | SlDDP10 | CGTCA-motif | CGTCA | 297 | 302 | 5 | - | *Hordeum vulgare* | *cis-*acting regulatory element involved in the MeJA-responsiveness |
| Solyc08g076310 | SlDDP10 | CGTCA-motif | CGTCA | 1096 | 1101 | 5 | + | *Hordeum vulgare* | *cis-*acting regulatory element involved in the MeJA-responsiveness |
| Solyc08g076310 | SlDDP10 | CGTCA-motif | CGTCA | 1950 | 1955 | 5 | + | *Hordeum vulgare* | *cis-*acting regulatory element involved in the MeJA-responsiveness |
| Solyc08g076310 | SlDDP10 | ABRE | ACGTG | 941 | 946 | 5 | + | *Arabidopsis thaliana* | *cis-*acting element involved in the abscisic acid responsiveness |
| Solyc08g076310 | SlDDP10 | P-box | CCTTTTG | 400 | 407 | 7 | + | *Oryza sativa* | gibberellin-responsive element |
| Solyc08g076310 | SlDDP10 | TGACG-motif | TGACG | 297 | 302 | 5 | + | *Hordeum vulgare* | *cis-*acting regulatory element involved in the MeJA-responsiveness |
| Solyc08g076310 | SlDDP10 | TGACG-motif | TGACG | 1096 | 1101 | 5 | - | *Hordeum vulgare* | *cis-*acting regulatory element involved in the MeJA-responsiveness |
| Solyc08g076310 | SlDDP10 | TGACG-motif | TGACG | 1950 | 1955 | 5 | - | *Hordeum vulgare* | *cis-*acting regulatory element involved in the MeJA-responsiveness |
| Solyc08g076310 | SlDDP10 | AuxRR-core | GGTCCAT | 955 | 962 | 7 | - | *Nicotiana tabacum* | *cis-*acting regulatory element involved in auxin responsiveness |
| Solyc09g064810 | SlDDP11 | TATC-box | TATCCCA | 1361 | 1368 | 7 | + | *Oryza sativa* | *cis-*acting element involved in gibberellin-responsiveness |
| Solyc09g064810 | SlDDP11 | CGTCA-motif | CGTCA | 508 | 513 | 5 | - | *Hordeum vulgare* | *cis-*acting regulatory element involved in the MeJA-responsiveness |
| Solyc09g064810 | SlDDP11 | P-box | CCTTTTG | 378 | 385 | 7 | + | *Oryza sativa* | gibberellin-responsive element |
| Solyc09g064810 | SlDDP11 | AuxRR-core | GGTCCAT | 931 | 938 | 7 | - | *Nicotiana tabacum* | *cis-*acting regulatory element involved in auxin responsiveness |
| Solyc09g064810 | SlDDP11 | TGACG-motif | TGACG | 508 | 513 | 5 | + | *Hordeum vulgare* | *cis-*acting regulatory element involved in the MeJA-responsiveness |
| Solyc12g088230 | SlDDP12 | LTR | CCGAAA | 1013 | 1019 | 6 | + | *Hordeum vulgare* | *cis-*acting element involved in low-temperature responsiveness |
| Solyc12g088230 | SlDDP12 | ABRE | CACGTG | 865 | 871 | 6 | + | *Arabidopsis thaliana* | *cis-*acting element involved in the abscisic acid responsiveness |
| Solyc12g088230 | SlDDP12 | ABRE | ACGTG | 866 | 871 | 5 | + | *Arabidopsis thaliana* | *cis-*acting element involved in the abscisic acid responsiveness |
| Solyc12g088230 | SlDDP12 | ERE | ATTTTAAA | 134 | 142 | 8 | + | *Nicotiana glutinos* | Ethylene Responsive |
| Solyc12g088230 | SlDDP12 | ERE | ATTTTAAA | 226 | 234 | 8 | + | *Nicotiana glutinos* | Ethylene Responsive |
| Solyc12g088230 | SlDDP12 | ERE | ATTTTAAA | 1789 | 1797 | 8 | + | *Nicotiana glutinos* | Ethylene Responsive |
