# Supplementary material for: The DUF221 domain-containing (DDP) genes identification and expression analysis in tomato under abiotic and phytohormone stress
Source: GM Crops Food. 2021 Aug 11;12(1):586–99. doi: 10.1080/21645698.2021.1962207 (PMC8820248; doi:10.1080/21645698.2021.1962207)
Supplement: Supplemental Material [file KGMC_A_1962207_SM7381.zip › supplementary/Table S7.docx]

| **Table S7. List of miRNAs predicted by psRNATarget to target the DDP genes of tomato SlDDPs** | | | | | | | | | | | | |
| --- | --- | --- | --- | --- | --- | --- | --- | --- | --- | --- | --- | --- |
| miRNA_Acc. | Target_Acc. | Expectation | UPE | miRNA_start | | miRNA_end | Target_start | Target_end | miRNA_aligned_fragment | Target_aligned_fragment | Inhibition | Multiplicity |
| sly-miR395a | *SlDDP3* | 4.5 | 14.894 | | 1 | 22 | 578 | 599 | CUGAAGUGUUUGGGGGAACUCC | CUGGUUUCCUUGGAUGCUUUGG | Cleavage | 1 |
| sly-miR395b |  | 4.5 | 14.894 | | 1 | 22 | 578 | 599 | CUGAAGUGUUUGGGGGAACUCC | CUGGUUUCCUUGGAUGCUUUGG | Cleavage | 1 |
| sly-miR171b |  | 5 | 15.964 | | 1 | 21 | 226 | 246 | UUGAGCCGUGCCAAUAUCACG | ACUCAUAUUGUUAUGGCUUAU | Translation | 1 |
| sly-miR319b | *SlDDP5* | 5 | 20.139 | | 1 | 21 | 1600 | 1620 | UUGGACUGAAGGGAGCUCCCU | UUUGAGGUCCCUUCAGUUCCU | Cleavage | 1 |
| sly-miR6022 |  | 5 | 22.923 | | 1 | 21 | 283 | 303 | UGGAAGGGAGAAUAUCCAGGA | UUCCGAAUAUUCUCCAUUGCA | Cleavage | 1 |
| sly-miR6026 | *SlDDP7* | 4.5 | 8.915 | | 1 | 22 | 1625 | 1646 | UUCUUGGCUAGAGUUGUAUUGC | AAAAUACAUUUCUAGUAAAGAC | Cleavage | 1 |
| sly-miR6022 | *SlDDP10* | 5 | 16.587 | | 1 | 21 | 1304 | 1324 | UGGAAGGGAGAAUAUCCAGGA | UCUUGUUUGUUCUUCCUGCCA | Cleavage | 1 |
| sly-miR482a | *SlDDP11* | 5 | 22.191 | | 1 | 22 | 1906 | 1927 | UUUCCAAUUCCACCCAUUCCUA | UUUAAAGGUGUGGAGUUGGACA | Cleavage | 1 |
