# Supplementary figures and images for: The DUF221 domain-containing (DDP) genes identification and expression analysis in tomato under abiotic and phytohormone stress
Source: GM Crops Food. 2021 Aug 11;12(1):586–99. doi: 10.1080/21645698.2021.1962207 (PMC8820248; doi:10.1080/21645698.2021.1962207)

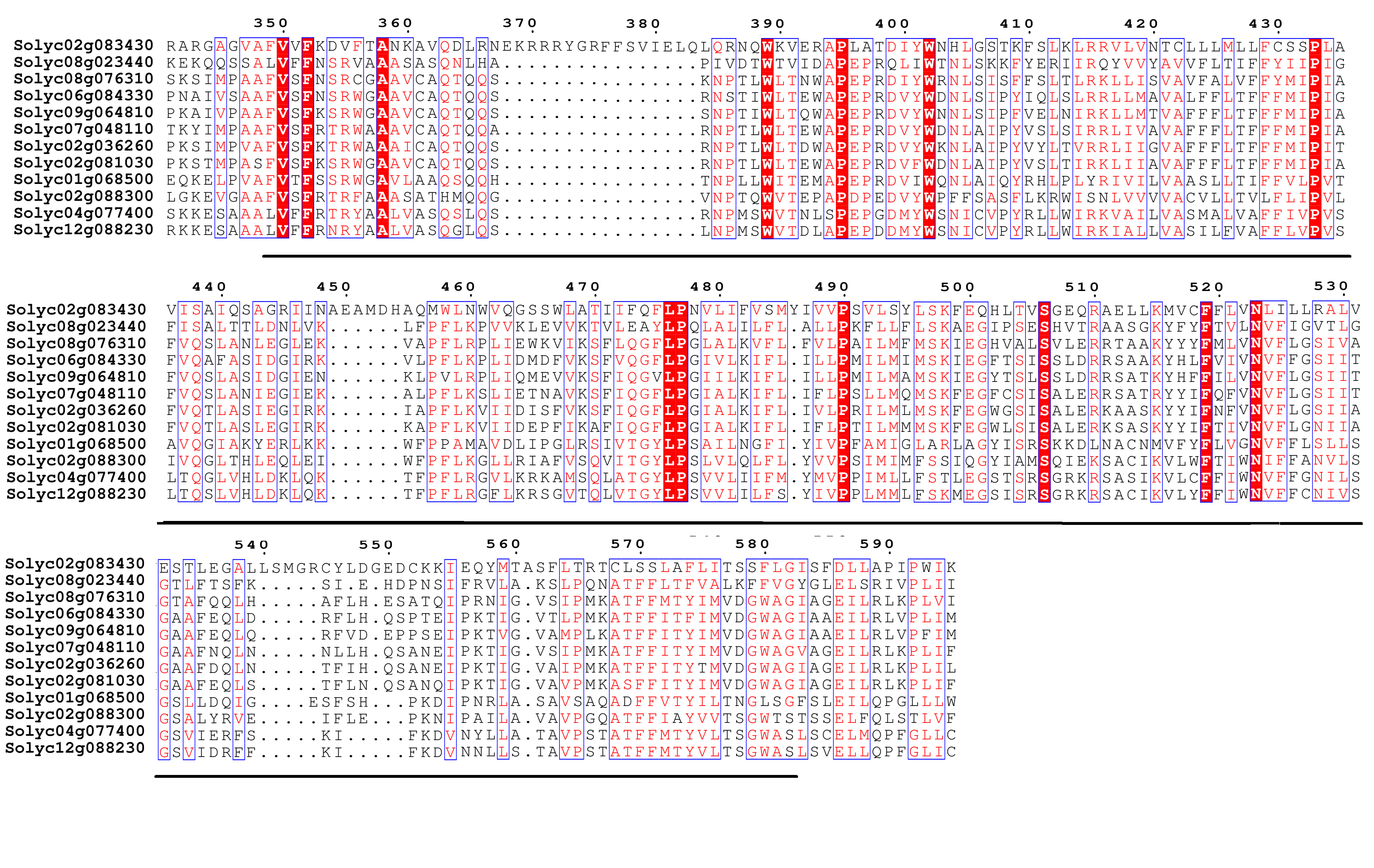

Supplement: Supplemental Material [file KGMC_A_1962207_SM7381.zip › supplementary/Fig S1.tif]
